# Non-Invasive Colorimetric Magneto Loop-Mediated Isothermal Amplification (CM-LAMP) Method for *Helicobacter pylori* Detection

**DOI:** 10.4014/jmb.2101.01008

**Published:** 2021-02-25

**Authors:** Khotchawan Bangpanwimon, Pimonsri Mittraparp-arthorn, Kanchana Srinitiwarawong, Natta Tansila

**Affiliations:** 1 Department of Microbiology, Division of Biological Science, Faculty of Science, Prince of Songkla University, Hat-Yai, Songkhla 90110, Thailand; 2 Molecular Evolution and Computational Biology Research Unit, Faculty of Science, Prince of Songkla University, Hat-Yai, Songkhla 90110, Thailand; 3 Faculty of Medical Technology, Prince of Songkla University, Hat-Yai, Songkhla 90110, Thailand

**Keywords:** Loop-mediated isothermal amplification (LAMP), colorimetric magneto LAMP assay (CM-LAMP), magnetic nanoparticles (MNPs), *Helicobacter pylori*, non-invasive test, saliva

## Abstract

More than half the world’s population is thought to be infected with *Helicobacter pylori*. Although the majority of infected people are asymptomatic, *H. pylori* infection may cause gastric ulcers and deadly gastric cancer. Owing to the difficulty and invasiveness of current routine culture and diagnostic methods, a highly sensitive and specific noninvasive assay for *H. pylori* is of interest. This study highlighted the design and performance of a colorimetric magneto loop-mediated isothermal amplification (CM-LAMP) assay to detect *H. pylori* in spiked saliva samples. LF primers were coated on magnetic nanoparticles by carbodiimide-induced immobilization and functionally used for solidphase amplification. During the LAMP reaction at 66°C, biotin-tagged FIPs were incorporated into LAMP amplicons. The colorimetric signal developed after the addition of NeutrAvidin horseradish peroxidase conjugate (NA-HRP) and ABTS. None of the tested microorganisms, including closely related bacteria, was shown positive by the CM-LAMP assay except *H. pylori* isolates. This novel platform was highly specific and 100-fold more sensitive (40 CFU/ml or 0.2 CFU per reaction) than the PCR and conventional LAMP assays for the detection of *H. pylori* in spiked saliva. Our results demonstrated the feasibility of using this noninvasive molecular diagnostic test to detect *H. pylori* in saliva samples.

## Introduction

*Helicobacter pylori* is one of the bacterial species classified by the International Agency for Research on Cancer (IARC) as a Group 1 carcinogen [1]. This bacterium is a causative agent of peptic ulcer and gastric cancers. It is well adapted to the human organism and has been isolated from more than half the people in the world. The route of *H. pylori* transmission remains questionable but the most probable paths are the oral-oral route via saliva and vomit and the fecal-oral route [2].

The techniques for *H. pylori* detection can be divided into two categories depending on the use of endoscopy for gastric biopsy collection. Histology, culture, and the rapid urease test (RUT) are considered invasive methods, while the urea breath test (UBT), the stool antigen test (SAT), serological testing, and molecular techniques that use samples of saliva or feces are considered noninvasive methods [3].

Invasive methods require experienced and skilled personnel, and detection depends heavily on the concentration of microorganisms. Also, several oral and gastric flora, such as *Proteus mirabilis*, *Klebsiella pneumoniae*, and *Staphylococcus aureus*, exhibit urease activity and can generate false-positive results in the RUT and UBT [4, 5]. Moreover, *H. pylori* becomes viable but not culturable (VBNC) in non-suitable conditions, leading to false-negative results [6, 7]. Also, the efficiency of antigen detection by the SAT was adversely affected as a result of diluted targets in watery stool samples [8]. The serology test has lower specificity and sensitivity than other methods and its positivity may not indicate a current active infection [9]. Although several molecular techniques, mostly based on PCR, have been reported, they often had problems of limited sensitivity resulting from PCR inhibitors as well as low numbers of *H. pylori*, especially in stool and saliva samples [10, 11].

**Fig. 1:**
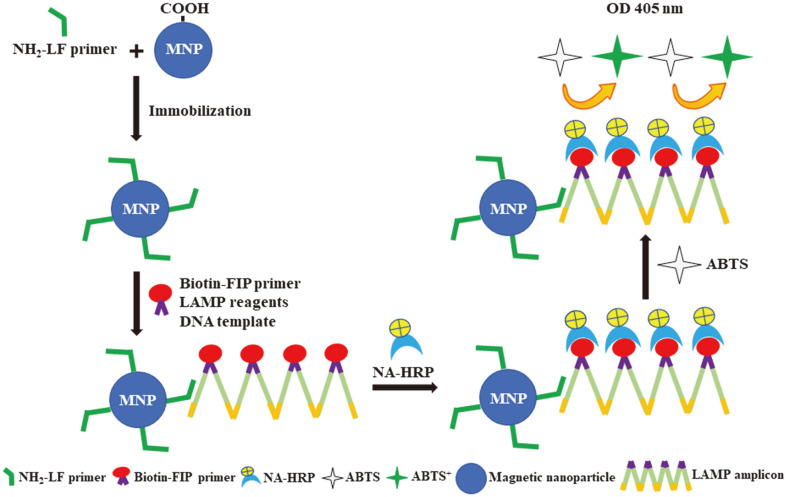
Schematic diagram of the colorimetric magneto-LAMP (CM-LAMP) assay. The carboxylated magnetic nanoparticles (MNPs) were immobilized with the amino-modified LF primer (NH_2_-LF primer) using the carbodiimide method. During amplification at 66°C, the MNP-conjugated LF primer annealed and amplified the target gene, and the biotinlabeled LAMP amplicons were then produced. After that, the amplicons were mixed with NeutrAvidin- horseradish peroxidase conjugate (NA-HRP), and an ABTS (colorless) substrate was subsequently added to generate the blue-green ABST^+^ products. The optical density (OD) of the colorimetric signal was measured at 405 nm.

Bearing in mind the difficulties of cultivation and the invasiveness of currently-used diagnostic procedures, a sensitive and specific noninvasive method of detecting *H. pylori* in saliva samples is of interest. In this work we aimed to improve the efficacy of analysis by incorporating the known advantages of loop-mediated isothermal amplification (LAMP), magnetic nanoparticles (MNPs), and a colorimetric system. The low abundance of *H. pylori* in saliva was overcome by the specificity and high sensitivity of LAMP [12]. A colorimetric system was previously used to increase the accuracy and reliability of LAMP product measurement [13] and the selective enrichment of LAMP products was facilitated by the superparamagnetism and biocompatibility of MNPs [14, 15]. Against this background, we present for the first time the design of an MNP-LAMP-colorimetric system, the so-called colorimetric magneto (CM)-LAMP assay, to detect low numbers of *H. pylori* in saliva. A schematic diagram of the novel system was presented in Fig. 1.

## Materials and Methods

### Isolation, Identification, and Confirmation of *H. pylori*

This study was approved by the Office of Human Research Ethics Committee (HREC), Prince of Songkla University (EC: 58-241-19-2). Between August 2015 and June 2016, residual gastric biopsy specimens were obtained from dyspepsia patients at the NKC Institute of Gastroenterology and Hepatology, Songklanagarind Hospital, Prince of Songkla University. The samples were transferred into brain heart infusion (BHI) transport media with 2,500 IU/L polymyxin B, 5 μg/ml trimethoprim, and 10 μg/ml each of vancomycin and amphotericin B. They were inoculated on Columbia agar supplemented with 7% sheep blood (Oxoid, UK) and incubated at 37°C under microaerophilic conditions for 3-7 days. *H. pylori* was identified based on colony morphology (0.5-1.5 mm pinpoint and translucent with slight hemolysis) and biochemical testing (urease-, catalase-, and oxidase-positive). Finally, the typical colony was subjected to confirmation by PCR assay to amplify a 294-bp fragment of the housekeeping *glmM* genes of *H. pylori* as previously described [16].

### LAMP Assay

**LAMP primer design**. The DNASTAR software was used to perform multiple alignments of *16S rRNA* sequences of 10 *H. pylori* strains (26695, J99, OK310, SJM180, v225d, HPAG1, XZ274, 51, 908 and 2018) against those of *H. mustelae*, *H. heilmannii*, *W. succinogenes*, *C. jejuni*, *C. coli*, *P. mirabilis*, *U. urealyticum*, *K. pneumoniae*, *M. morganii*, *N*, *meningitidis*, *E. coli*, *V. parahaemolyticus*, and *V. cholerae*. Using the PrimerExplorer v4.0 software, a set of LAMP primers was designed to selectively target a highly conserved region (Table 1). The obtained primers were analyzed by the nucleotide BLAST tool to ensure specificity, and primer-dimer and hairpin loop formation were checked by vector NTI software.

**LAMP condition**. The LAMP assay was performed in a final volume of 25 μl comprising 5 μl of extracted DNA, 0.25 μM of primer F3, 0.2 μM of primer B3, 1.6 μM of primers FIP and BIP, 0.8 μM of primers LF and LB, 8 U of *Bst* DNA polymerase (New England Biolabs, USA), 0.8 M betaine solution (Sigma-Aldrich, USA), 8 mM MgSO_4_ and 1.4 mM dNTPs in LAMP 4 mix buffer (20 mM Tris–HCl, pH8.8), 10 mM KCl, 10 mM (NH_4_)_2_SO_4_, and 0.2%Tween 20. The reaction mixture was incubated in the Loopamp Realtime Turbidimeter (Eiken Chemical, Japan) for 35 min at 66°C and inactivated for 2 min at 80°C. The reaction was judged as positive when turbidity reached 0.1 within 35 min.

### Speciﬁcity and Sensitivity of the LAMP Assay in Pure Culture

The speciﬁcity of the designed LAMP primers was evaluated using pure cultures of *H. pylori*, *Campylobacter* spp., and urease-positive and pathogenic bacteria commonly found in the oral and enteric cavities (Table 2). A typical colony of each bacterium was boiled for 10 min in 300 μl of DI water. After centrifugation at 20,000 g, 4°C for 10 min, the supernatant was collected and used as the DNA template. For sensitivity testing, the *H. pylori* culture was adjusted to 10^7^ CFU/ml and tenfold dilution was performed to obtain a range of bacterial concentrations from 4 × 10^1^ to 4×10^5^ CFU/ml. Bacteria were enumerated by drop plate technique [17]. A PCR specific to the *glmM* gene was conducted and the results were compared with the results of LAMP. Concentration testing was performed in triplicate and the limit of detection (LOD) was described as the lowest bacterial concentration giving positive results in all three replicates.

### Sensitivity of the LAMP Assay in Spiked Saliva

Saliva from healthy individuals was collected and tested for the absence of *H. pylori* by *glmM*-specific PCR. Saliva confirmed free of the bacterium was then inoculated with *H. pylori* at ﬁnal concentrations ranging from 4×10^1^ to 4 × 10^5^ CFU/ml. DNA templates from spiked saliva were prepared by Presto Mini gDNA Bacteria Kit (Geneaid Biotech, Taiwan) and subjected to both *glmM*-specific PCR and LAMP assay for sensitivity analyses. Triplicate experiments were applied to all concentration testing and the LOD was obtained as described above.

### Colorimetric Magneto (CM)-LAMP Assay

**Immobilization of primers onto MNPs.** Carboxylated MNPs (Chemicell GmbH, Germany) were coated with NH_2_-modified LF primer using the carbodiimide method [18]. In brief, stock MNPs (25 mg/ml) were activated by sonication at 30% amplitude for 10 min. The activated MNPs (0.2 mg) were washed with 200 μl of 2-(N-morpholino)ethanesulfonic acid (MES) buffer (25 mM, pH 6.0) until the supernatant was clear. They were then mixed with NH_2_-modified LF primer (1-2.5 nmol) and incubated for 30 min at room temperature under gentle shaking, after which 16 μl of 1-ethyl-3-(3dimethylaminopropyl) carbodiimide (EDC) solution (10 mg/ml) were added. After overnight incubation at 4°C under gentle shaking, the supernatant was collected. Using the MaestroNano spectrophotometer (Maestrogen, Taiwan), the absorbance of the supernatant was measured at 260 nm, which represented the residual concentration of NH_2_-modified LF primer. Following a previously described method [19], the concentration of immobilized LF primer on MNPs (Q value, nmol of primers/mg of MNPs) was determined and calculated from the formula Q = [(C_o_ – C_e_) × V]/(M × m), where Q is the primer immobilization efficiency (nmol primer/mg MNPs), C_o_ and C_e_ are the initial and residual primer concentrations (ng/μl), M is the molecular weight of oligonucleotide (ng/nmol), m is the mass of MNPs (mg), and V is the volume of solution (μl). The primer-immobilized MNPs were mixed with Tris buffer (50 mM, pH 7.4) to quench the unreacted carboxyl groups. The quenching step was repeated twice. Finally, the primer-immobilized MNPs were re-suspended in 100 μl of Tris-EDTA buffer (10 mM Tris-HCl and 1 mM EDTA, pH 8.0) and stored at 4°C until used.

**Immobilization efficiency testing of primer-coated MNPs.** The efficiency of LF primer-coupled MNPs was analyzed by the hybridization technique previously indicated [20]. Briefly, 5 μl of LF primer-immobilized MNPs (2 μg/μl) was washed twice with 100 μl of phosphate-buffered saline (PBS) buffer (pH 7.4). Then, 2 μl of 10 μM complementary LF primer tagged with biotin (5'-biotin-GAAGGTGGGGACGACGTCAA-3') in 13×saline-sodium citrate (SSC) buffer was added and the MNPs were incubated at RT for 30 min under gentle shaking. The MNPs were washed twice with 150 μl of PBS containing 1% bovine serum albumin (BSA) and then incubated in darkness with 2.5 μl of NeutrAvidin horseradish peroxidase conjugate (NA-HRP) (0.01 mg/ml) (Thermo Scientific, USA) in PBS buffer at RT under gentle shaking for 30 min. After washing twice with PBS buffer, 50 μl of 2,2′-Azino-bis (3-ethylbenzthiazoline-6-sulfonic acid) (ABTS) (Merck KGaA, Germany) containing H_2_O_2_ in a citrate buffer (pH 4.0) was added and incubated in darkness at RT under gentle shaking for 30 min. HRP activity was measured spectrophotometrically at 405 nm using a microplate reader (BMG Labtech, Germany). DI water and uncoupled MNPs were used as negative controls.

### CM-LAMP Assay

The CM-LAMP assay was performed in 25 μl containing 5 μg of LF primer-immobilized MNPs, 0.25 μM of primer F3, 0.2 μM of primer B3, 0.8 μM of primer LB, 1.6 μM of primer BIP and biotin-labeled FIP (Table 1), 5 μl of extracted DNA, 8 U of *Bst* DNA polymerase, and 12.5 μl of 2× Reaction Mix Loopamp DNA Amplification Kit (Eiken Chemical, Japan). The reaction mixture was incubated in the Loopamp Realtime Turbidimeter for 50 min at 66°C and was then inactivated for 2 min at 80°C. MNP-LAMP-Biotin products were measured using a colorimetric end-point system. Reactions were treated with 150 μl of 1% BSA solution for 1 h at 37°C and incubated in darkness with 2.5 μl of NA-HRP (0.01 mg/ml) in PBS buffer at RT under gentle shaking for 30 min. After washing the reaction products twice with PBS buffer, 50 μl of ABTS (containing H_2_O_2_) in a citrate buffer (pH 4.0) was added and the products were incubated in darkness at RT under gentle shaking for 30 min. HRP activity was measured at 405 nm using the microplate reader.

### Speciﬁcity and Sensitivity of CM-LAMP Assay in Pure Culture and Spiked Saliva

The speciﬁcity of the CM-LAMP assay was evaluated using pure cultures of 19 clinical isolates of *H. pylori*, *Campylobacter* spp., urease-positive bacteria, and commensal bacteria. The sensitivity of the CM-LAMP assay was evaluated using the extracted DNA template from pure cultures and spiked saliva using the methods described previously. The cut-off value of a positive result was derived from the formula NC + 3SE in which NC and SE are the averages and standard error of OD_405nm_ from negative controls [20, 21]. The SE of the mean values was shown as a thin line above the bars.

## Results

### Characteristics of *H. pylori* Isolates

A total of 50 gastric biopsy samples were collected from Songklanagarind Hospital. Seven isolates of *H. pylori* from dyspepsia patients were identified by biochemical tests and from *glmM*-specific PCR and used in primer specificity testing. One of the isolates was sequenced for 16s rRNA gene and used as an experimental strain in sensitivity testing. It possessed 98% identity to *H. pylori* strain ML2 at E-value 0 (data not shown).

### Conventional LAMP and PCR Assays

In this study, LAMP primers were designed based on a unique region of the *16S rRNA* gene of *H. pylori* (Table 1). The evaluation of LAMP specificity was based on DNA samples isolated from pure cultures of 7 isolates of *H. pylori*, 5 isolates of *Campylobacter jejuni*, 2 isolates of *Campylobacter coli*, 1 isolate of *Campylobacter lari*, and 15 isolates of urease-positive and pathogenic bacteria found in the oral and enteric cavities. As demonstrated in Table 2, all *H. pylori* isolates could be detected by our designed LAMP primers, while other isolates generated true negative results. LAMP detected *H. pylori* as low as 2 CFU per reaction or 400 CFU/ml in pure culture, which was 10-fold lower than the quantity detected by PCR (20 CFU per reaction or 4,000 CFU/ml in pure culture) (Table 3). Both LAMP and PCR assays gave consensus positivity for detection of 20 CFU of *H. pylori* in spiked saliva,(Table 3). It is worth noting that one-third of triplicate experiments of LAMP and PCR assays were positive at 2 CFU per reaction assay in both spiked saliva and pure culture (Table 3).

### CM-LAMP Assay

**Fig. 2:**
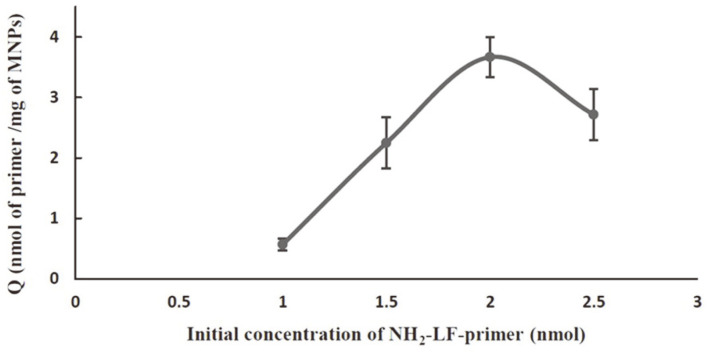
The initial concentration of NH_2_-modified LF primers immobilized on MNPs was varied from 1 nmol to 2.5 nmol. The MNPs (0.2 mg) were mixed with the primer concentration and incubated at 4°C overnight. The supernatant was collected to measure the residual concentration of the primers. The Q value of the immobilized LF primers on MNPs was determined from the following formula: Q = [(C_o_ – C_e_) × V]/(M × m) where Q is the efficiency of the primer immobilization (nmol primer/mg MNPs), C_o_ and C_e_ are the initial and residual primer concentrations (ng/μl), M is the molecular mass of the primer (ng/nmol), m is the mass of MNPs (mg), and V is the volume of solution (μl).

**Fig. 3:**
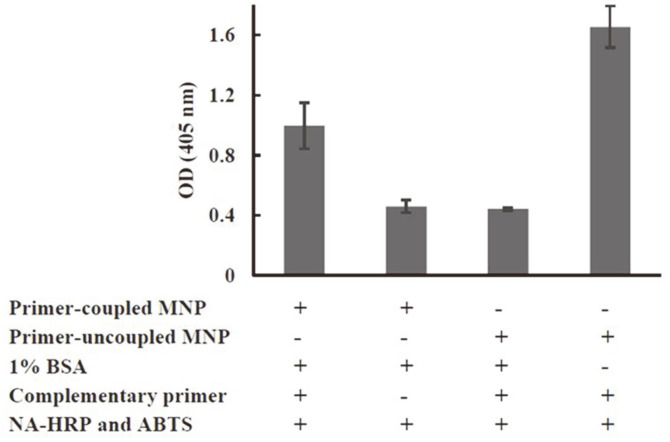
The hybridization of the biotinylated-complementary oligonucleotide to LF primer was used to analyze the efficiency of LF primer immobilization on the surface of MNPs. Bovine serum albumin (1% BSA) was added to reduce the non-specific adsorption of complementary primer on the surface of MNPs. After the addition of enzyme conjugate and substrate, the colorimetric signal of ABTS^·+^ was measured at 405 nm. Reactions mixed with DI water or primeruncoupled MNPs were recorded to evaluate the background absorbance. All experiments were done in triplicate and the means and SE values were plotted.

**Preparation and assessment of primer-immobilized MNPs.** LF primers with 5'-NH_2_ modification were immobilized onto the MNP surface by the carbodiimide method. After the coating steps, the absorbance of the supernatant was recorded at 260 nm. The amount of NH_2_-modified LF primer to be immobilized on the surface of the MNPs (Q)(nmol/mg) was calculated. Increasing the initial concentration of NH_2_-modified LF primers up to 2 nmol resulted in a higher Q value (Fig. 2). However, the Q value decreased when the initial concentration of the LF primer reached 2.5 nmol. To obtain efficient DNA amplification, the primer-immobilized MNPs prepared from an initial concentration of 2 nmol NH_2_−modified LF were employed in subsequent CM-LAMP assays. Thus, the obtained reagent had an LF primer density of 3.66 ± 0.33 nmol/mg MNPs (Fig. 2). A hybridization approach was used to ensure the completion of the primer-coupling reaction and the presence of LF primers on the surface of the MNPs. The biotinylated complementary primer could anneal to LF primers coated on the MNPs and give rise to an HRP-catalyzed colorimetric reaction (Fig. 3). The absorbance of this reaction was approximately 2-fold higher than that of the reaction without the complementary primer. The highest absorbance was found when uncoupled MNPs were used. This finding indicated the non-specific adsorption of MNPs, which was overcome by the addition of a blocking solution (1% BSA), as illustrated in Fig. 3. Therefore, these findings indicated that the LF-immobilized MNPs and other colorimetric reagents were possibly functional in the CM-LAMP assay for the detection of *H. pylori*.

**Fig. 4:**
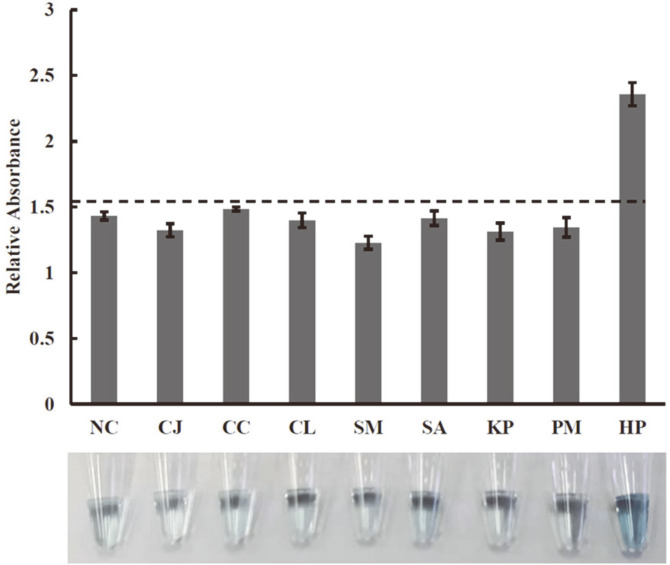
The chart illustrates the specificity of the CM-LAMP assay. The upper panel is a bar chart of relative absorbance and the lower panel presents pictures of corresponding reaction tubes. DNA extracted from pure cultures of oralenteric bacteria was tested by the CM-LAMP assay. DI water was used as a negative control (NC). All experiments were done in triplicate. The standard error (SE) of the mean values of each sample was shown above each bar. The cut-off value was obtained by measuring the relative OD_405nm_ of the negative control at 405 nm, and the average absorbance and 3SE were calculated to establish the cut-off value illustrated as a dashed line (NC + 3SE). NC = Negative control, CJ = *Campybacter jejuni*, CC = *Campybacter coli*, CL = *Campybacter lari*, SM = *Streptococcus mutans*, SA = *Staphylococcus aureus*, KP = *Klebsiella pneumoniae*, PM = *Proteus mirabilis*, HP = *Helicobacter pylori*.

**Fig. 5:**
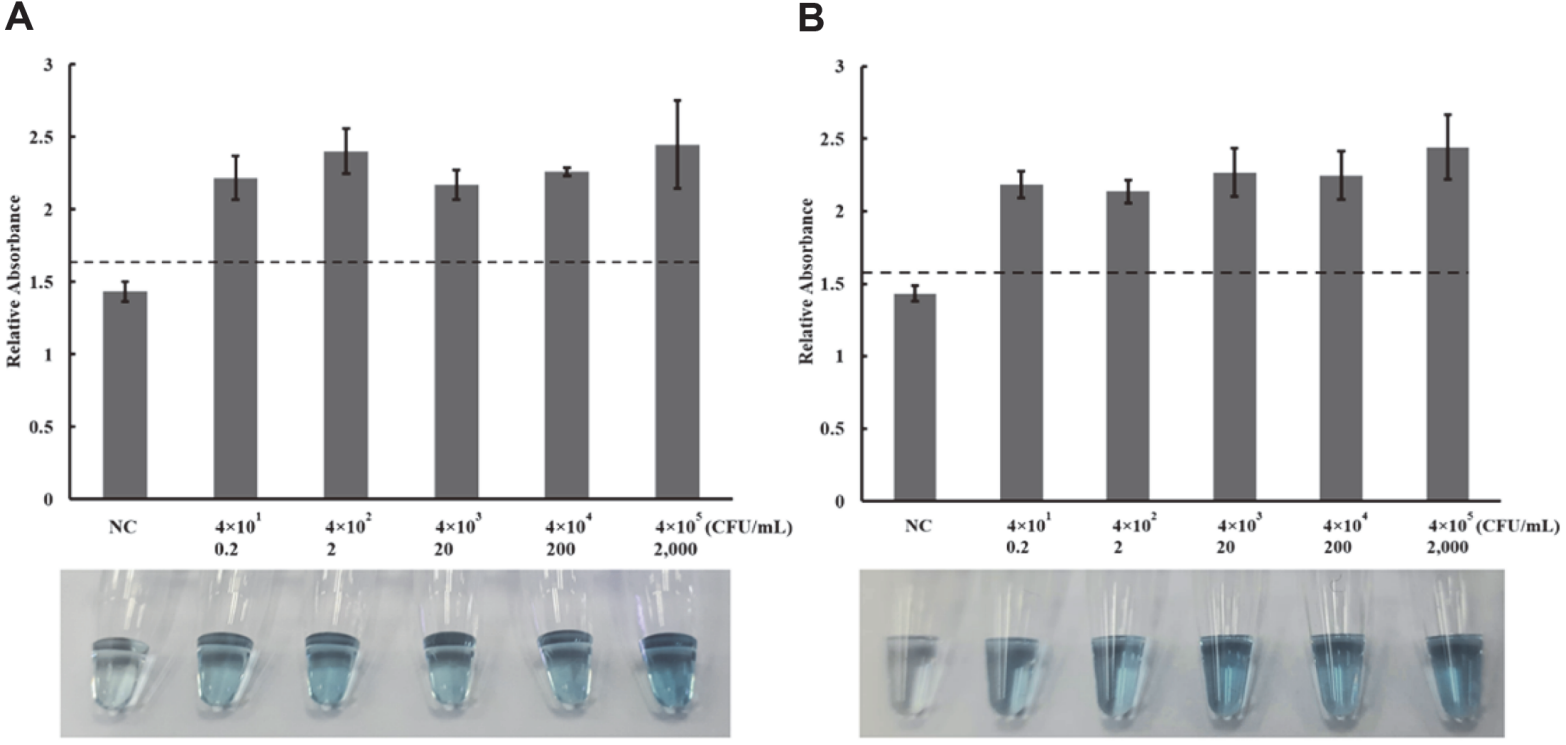
The charts illustrate the sensitivity of the CM-LAMP assay in pure culture (A) and spiked saliva (B). The upper panels are bar charts of relative absorbance, the lower panels present pictures of reaction tubes. Various DNA concentrations of *H. pylori* (4 × 10^1^ to 4 × 10^5^ CFU/ml) from pure cultures (**A**) or spiked saliva samples (**B**) were analyzed by the CM-LAMP assay. DI water or non-inoculated saliva was used as a negative control (NC). All experiments were done in triplicate. The cut-off value was obtained by measuring the relative OD_405nm_ of the corresponding negative control at 405 nm, and the average absorbance and 3SE were calculated to establish the cut-off value, illustrated as a dashed line (NC + 3SE).

**Efficiency of CM-LAMP assay for *H. pylori* detection in pure culture and spiked saliva.** The specificity of the CM-LAMP assay was determined using extracted genomic DNA of 19 clinical isolates of *H. pylori*, *Campylobacter* spp., urease-positive and commensal bacteria. The cut-off value was obtained from the determination of the reactions with no genomic DNA and calculated by the NC + 3SE formula mentioned earlier. The data in Fig. 4 revealed that true-positive results were obtained only from *H. pylori* isolates (relative absorbance above cut-off value), and neither false-positive nor false-negative results were obtained from the CM-LAMP assay. The genomic DNA of *H. pylori* was 10-fold serially diluted and analyzed by CM-LAMP assay. Fig. 5A showed that the relative OD_405nm_ values for *H. pylori* were 1.43 ± 0.07, 2.22 ± 0.15, 2.40 ± 0.15, 2.17 ± 0.10, 2.26 ± 0.03, and 2.45 ± 0.30. These values are equivalent to 0, 0.2, 2, 20, 200 and 2,000 CFU per reaction, respectively. These were all above the cut-off value of 1.64 (dash line) indicating that the LOD of the CM-LAMP assay in pure culture was 40 CFU/ml or 0.2 CFU per reaction, which is 100× and 10× lower than the LOD of PCR and conventional LAMP assays, respectively. Saliva from healthy individuals was artificially spiked with *H. pylori* before DNA extraction. CM-LAMP assay successfully detected *H. pylori* (Fig. 5B). At the end of measurement, reaction mixtures containing 0, 0.2, 2, 20, 200 and 2,000 CFU of *H. pylori* had relative absorbances of 1.43 ± 0.05, 2.18 ± 0.09, 2.14 ± 0.08, 2.27 ± 0.17, 2.25 ± 0.17, and 2.44 ± 0.22, respectively. In the context of a saliva matrix, the cut-off value was 1.58, and the LOD was 40 CFU/ml or 0.2 CFU per reaction, which is 100× lower than the LOD of PCR and conventional LAMP assays.

## Discussion

In this study, newly designed primers targeting the 16S rRNA gene were used for the specific detection of *H. pylori* by conventional LAMP and CM-LAMP assays. No other bacteria could produce a positive result in both LAMP assays, indicating the notable specificity (100%) of the assays (Table 2 and Fig. 4). This was mainly due to the function of two primer pairs (inner and loop primers) that selectively annealed on particular regions of the DNA template and then produced amplicons (Table 1). Inner primers (FIP and BIP) offer auto-cycling amplification through self-priming and self-extension of stem-loop DNA products. Meanwhile, loop primers (LF and LB) bind to a nucleotide loop created by the incorporation of inner primers and facilitate the additional extension of the targeted strand [22]. In Table 3, the better detection (10 times lower LOD) of conventional LAMP compared to PCR has been attributed to the continuously isothermal strand extension and high rate of product formation catalyzed by *Bst* DNA polymerase [23]. This also helped shorten operation time to less than an hour. Our conventional LAMP had the LOD of 400 CFU/ml which is approximately 10-to-100 times lower than LODs reported previously by two independent groups (2 × 10^3^ and 2 × 10^4^ CFU/ml) [24, 25]. Although all LAMP assays were specific to the *glmM* gene of *H. pylori*, our conventional LAMP was more sensitive, presumably because of the larger number of amplicons produced by the action of the two additional loop primers (LF and LB) [22]. The conventional LAMP assay has several notable advantages over the PCR assay: shorter operation time, cost-effective instrumentation, no requirement of post-reaction handling steps, and visible amplified products [26]. However, difficulties such as subjective interpretation (turbidity of Mg pyrophosphate and color change of HNB), inhibition of amplification (SyBr Green I), and exposure to a carcinogen (EtBr staining in gel electrophoresis) have been reported in the detection of LAMP products [27]. The multiple primers used in the LAMP reaction may increase the likelihood of primer-dimer and primer–primer hybridizations, possibly leading to false-positive results [27]. These problems reduce the feasibility of using LAMP in a routine laboratory setting. Therefore, to improve the efficiency of analysis in this study, CM-LAMP was developed by incorporating the advantages of LAMP, MNPs, and a colorimetric system. Applying an external magnetic force to MNPs enables simple isolation or concentration of target molecules. The large surface area of MNPs could provide a platform for bioreceptor immobilization for particular purposes [14]. In this work, LF primer-coupled MNPs were prepared using an initial amount of 2 nmol of NH_2_-modified oligonucleotides. Additionally, a long spacer (-C_12_-) was incorporated into the primer in order to minimize the steric hindrance effect during primer immobilization on the MNP surface and primer annealing to the DNA target [20]. However, a higher amount of primers (2.5 nmol) was used in the immobilization step and possibly caused electrostatic repulsion or steric hindrance at the surface of MNPs [28], which could negatively affect immobilization efficiency (Fig. 2). The prepared LF-coupled MNPs were verified by the addition of complementary biotinylated oligonucleotides and an HRP-ABTS mixture. Results indicated that the coated LF primers were accessible to their complementary counterpart and a colorimetric reaction ensued. Meanwhile, blocking the MNP surface with BSA was necessary to eliminate non-specific adsorption of complementary nucleotide sequences as well as false-positive signals (Fig. 3) [29]. The background absorbance observed when LF primer or complementary oligonucleotide was absent was probably due to ABTS oxidation catalyzed by the MNPs (Fig. 3). The MNPs were made of magnetite (Fe_3_O_4_) which harbors peroxidase-like activity [30]. Our CM-LAMP assay was successful in selectively amplifying the target region and giving a colorimetric reaction that aided result interpretation (Fig. 4). This outcome indicated that modified FIP remained functional in primer annealing and strand-extension during the LAMP reaction and interacted with NA-HRP during colorimetric detection [31-33]. The NA tag was used because it could generate lower nonspecific binding than avidin and streptavidin. The biotin-binding activity of NA was about 14 μg/mg of protein which is close to the theoretical maximum activity [34]. The green color of the ABTS^·+^ product was stable for an hour and could be measured spectrophotometrically [35]. The establishment of cut-off value was done by negative samples to exclude all background absorbance.

Our CM-LAMP assay was specific solely to *H. pylori* isolates, while other microorganisms including closely related ones and plausible contaminants gave absorbances below the cut-off value (Fig. 4). From LOD results, the CM-LAMP assay was between 10 and 100 times more sensitive in the detection of pure *H. pylori* culture (40 CFU/ml) in comparison with the conventional LAMP and PCR assays, respectively (Fig. 5A). It has been reported that saliva and vomitus samples collected from patients contained 10^2^ to 10^3^ CFU/ml *H. pylori* [36, 37]. The CM-LAMP assay achieved comparable LODs (100 times lower than conventional LAMP and PCR assays) when spiked saliva samples were tested (Fig. 5B). This indicated that mucin, a LAMP inhibitor in saliva, did not interfere with the analytical efficiency of our designed platform [38]. Many colorimetric LAMP assays have been developed through combination with other analytical approaches to increase their detectability. These techniques, which include LAMP-enzyme linked immunosorbent assay (LAMP-ELISA) [13], magneto reverse transcription LAMP-based chemiluminescence assay (RT-LAMP-CL) [39], magneto LAMP-based electrochemical assay (EC-LAMP) [40], and probe-specific LAMP magneto assay [31], have employed a two-step detection approach for amplifying the target gene and then hybridizing to the specific capture probes coated on the surface of magnetic materials or a microtiter plate. Our CM-LAMP assay offers a novel platform using modified LF and FIP primers which enables simultaneous solid-phase amplification, hybridization, and amplicon labeling. This not only reduces the operation time but also provides more efficient strand extension-mediated amplicon capture between the labeled LAMP product and MNPs. The LOD of the LAMP-ELISA assay for *Salmonella enterica* ser. Paratyphi and serogroup D has been reported with the LOD of 10 and 10^3^ CFU/ml in spiked blood and spiked meat, respectively [13, 32]. The high sensitivity and specificity of the CM-LAMP assay affirm the feasibility of using this technique for noninvasive *H. pylori* detection in saliva samples.

In conclusion, we presented a non-invasive, rapid, and highly sensitive platform of a CM-LAMP assay to selectively detect *H. pylori* in saliva samples. This technique combines many advantages of the LAMP assay, magnetic nanoparticles, and a colorimetric system to attain high sensitivity (~1 CFU per reaction) and specificity. Exceptionally, our platform could provide a complete analysis in less than three hours and can also be applied to other target genes or microorganisms. This method could serve as a new approach for developing an automatic system for molecular diagnostics.

## Figures and Tables

**Table 1:** Primers used for conventional LAMP and CM-LAMP assays for *H. pylori* detection.

Primer name	Primer Sequence (5' to 3')	Length (bp)
16s HP-F3	TGCTAACAGGTYATGCTGAG	20
16s HP-B3	GCAACATGGCTGATTTGCG	19
16s HP-FIP^a^	TAGCCCTAGGCGTAAGGGCCCTGCCTCCGTAAGGAGGAG	39
16s HP-BIP	ACTGCGAAGTGGAGCCAATCTTGCTTCATGCAGGCGAGTT	40
16s HP-LF^b^	TTGACGTCGTCCCCACCTTC	20
16s HP-LB	ACACCTCTCAGTTCGGATTGTAGG	24

^a^For CM-LAMP assay, 16s HP-FIP primer was labeled with biotin at 5' end.

^b^For CM-LAMP assay, 16s HP-LF primer was modified with NH_2_-C_12_ at 5'end.

**Table 2:** Specificity of *H. pylori* detection by conventional LAMP assay.

Bacteria	No. of isolates	Conventional LAMP assay	

		+	-
Campylobacterales			
*Helicobacter pylori*	7	7	0
*Campybacter jejuni*	5	0	5
*Campybacter coli*	2	0	2
*Campybacter lari*	1	0	1
Oral bacteria			
*Streptococcus mutans* DMST 1877	1	0	1
Urease +ve bacteria			
*Staphylococcus aureus* DMST 20654	1	0	1
*Neisseria meningitidis*	1	0	1
*Klebsiella pneumoniae*	1	0	1
*Proteus mirabilis*	1	0	1
*Providensia rettgeri*	1	0	1
*Citrobacter freundii*	1	0	1
Enteric bacteria			
*Shigella boydii*	1	0	1
*Shigella flexneri*	1	0	1
*Aeromonas hydrophila*	1	0	1
*Salmonella* Paratyphi	1	0	1
*Salmonella* Enteritidis	1	0	1
*Vibrio paraheamolyticus*	1	0	1
*Vibrio cholerae* ATCC 39315	1	0	1
*Escherichia coli* ATCC 8739	1	0	1
*Pseudomonas aeruginosa* ATCC 27853	1	0	1

**Table 3:** Limit of *H. pylori* detection by PCR, LAMP and CM-LAMP assays.

Sample	Assay	Results (positive reactions/numbers tested)					

		4×10^5^	4×10^4^	4×10^3^	4×10^2^	4×10^1^	CFU/ml

		2×10^3^	2×10^2^	2×10^1^	2×10^0^	2×10^-1^	CFU/reaction
Pure culture							
	PCR	+	+	+	± (1/3)	-	
	LAMP	+	+	+	+	-	
	CM-LAMP	+	+	+	+	+	
Spiked saliva							
	PCR	+	+	+	-	-	
	LAMP	+	+	+	± (1/3)	-	
	CM-LAMP	+	+	+	+	+	

+ = Positive in all triplicate experiments.

- = Negative in all triplicate experiments.

± = Variable detection in triplicate experiments (positive reactions/numbers tested).
